# Therapeutic approaches and long-term follow-up for prenatal hydronephrosis

**DOI:** 10.12669/pjms.323.9133

**Published:** 2016

**Authors:** Bahattin Aydogdu, Gulay Tireli, Oyhan Demirali, Unal Guvenc, Cemile Besik, Serdar Sander, Aysel Kiyak

**Affiliations:** 1 Bahattin Aydogdu, M.D. Department of Pediatric Surgery, Kanuni Sultan Suleyman Research and Teaching Hospital, 34303, Istanbul, Turkey; 2 Gulay Tireli, Associate Professor, Department of Pediatric Surgery, Kanuni Sultan Suleyman Research and Teaching Hospital, 34303, Istanbul, Turkey; 3 Oyhan Demirali, M.D. Department of Pediatric Surgery, Kanuni Sultan Suleyman Research and Teaching Hospital, 34303, Istanbul, Turkey; 4 Unal Guvenc, M.D. Department of Pediatric Surgery, Kanuni Sultan Suleyman Research and Teaching Hospital, 34303, Istanbul, Turkey; 5 Cemile Besik, M.D. Department of Pediatric Surgery, Kanuni Sultan Suleyman Research and Teaching Hospital, 34303, Istanbul, Turkey; 6 Serdar Sander, Associate Professor, Department of Pediatric Surgery, Kanuni Sultan Suleyman Research and Teaching Hospital, 34303, Istanbul, Turkey; 7 Aysel Kiyak, M.D. Department of Pediatric Nephrology, Kanuni Sultan Suleyman Research and Teaching Hospital, 34303, Istanbul, Turkey

**Keywords:** Antenatal, Hydronephrosis, Pyeloplasty, Unilateral

## Abstract

**Objective:**

This study summarises the outcomes of 149 patients who underwent surgery for antenatally diagnosed unilateral hydronephrosis.

**Methods:**

The medical records of such patients over a 23-year period were reviewed retrospectively. Age at the time of operation, preoperative and postoperative mean pelvic diameter on ultrasound, split renal function, washout patterns on scintigraphy, and early and late complications were recorded.

**Results:**

The mean preoperative follow-up period was five months (range: 1–66 months). One patient was operated on after 12 months and two patients after five years of follow-up. Mean preoperative pelvic diameter and renal function were 30.8 mm and 38.6%, respectively; all patients had an obstructive wash-out pattern. In the postoperative period, the corresponding measurements were 11.7 mm and 39.2%, with 111 non-obstructive, 24 partially obstructive, and 14 obstructive wash-out patterns. Three patients with severe caliectasis and low renal function underwent surgery despite mild hydronephrosis. The mean postoperative follow-up period was six (range 4–11) years. Complications developed in 14 (9.3%) patients.

**Conclusion:**

Patients with antenatal hydronephrosis may need surgery even after a follow-up period of six years. Because of the potential late development of complications, postoperative follow-up should be continued for 10 years.

## INTRODUCTION

There is no consensus regarding the follow-up and surgical criteria for patients diagnosed with antenatal hydronephrosis (HN).^1^ Both prolonged, conservative follow-up^2^ and the aggressive use of surgery have been suggested. Advocates of the former recommend a follow-up period as long as 10 years.^3^ Among patients with antenatally diagnosed ureteropelvic junction (UPJ) obstruction who required long-term follow-up, only 22% eventually underwent surgery.^4^ The recommendations for post-surgical follow-up differ as well, ranging from 2 to 10 years.^2^,^3^,^5^

This report summarises the 23 years of experience gained in the surgical treatment of 149 HN patients. Preoperative and postoperative parameters, patient characteristics, and early and late results of treatment are discussed.

## METHODS

We retrospectively reviewed the medical records of 549 newborn patients admitted to our pediatric nephrology and pediatric surgery units for unilateral HN. Patients with ureterovesical obstruction were excluded. HN was classified according to the Society of Fetal Urology criteria (antero-posterior diameter and renal functions) (Table-I). The final study population consisted of 149 patients who underwent surgery for UPJ obstruction between 1987 and 2011.

**Table-I:** The data of patients in regard to SFU grade, SRF, treatment modality.

*SFU grade (n=549)*	*PD (mm)*	*Preop. SRF % (n)*	*Treatment*
SFU 1(181)	15-20	45-50	Conservative
SFU 2 (212)	20-30	40-45 (207) 40< (5)	Conservative Pyeloplasty
SFU 3 (104)	20-40 20-40 20-30	40-45 (12) 30-40 (73) <30 (19)	Conservative Pyeloplasty Pyeloplasty
SFU 4 (52)	>30 >40	20-40 (35) 10-20 (17)	Pyeloplasty Pyeloplasty

SFU: Society of fetal urology, Preop: Preoperative, PD: pelvic diameter, SRF: split renal function

**Fig.1:**
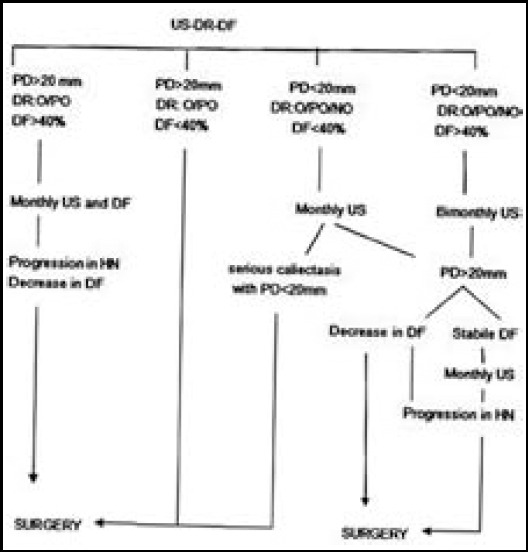
Follow-up and treatment algorithm for patients with antenatally diagnosed hydronephrosis. US: ultrasound, DR: diuretic renogram, DF: differential function, HN: Hydronephrosis, O: obstructive, PO: partially obstructive, NO: nonobstructive.

Informed consent was obtained from the patients’ parents. Patient age, preoperative and postoperative length of follow-up, antero-posterior pelvic diameter (PD) measurement, findings on ultrasound (US), 99mTc-DMSA renal SPECT, diuretic renogram (DTPA/MAG3) and vesico-cysto-urethrography (VCUG) as well as all relevant complications were recorded. Statistical analysis consisted of both paired and unpaired Student’s t-tests. The treatment algorithm is shown in Fig.1. A flank incision was used in patients undergoing Anderson-Hynes pyeloplasty. The procedure included the placement of a double-J ureteral catheter and a perirenal drainage catheter. The perirenal drain was removed on the seventh postoperative day and the double-J stents were removed after one month.

The patients were evaluated by Ultrasound on postoperative day 10 and after months one, three and six using our routine clinical protocol. In addition, the washout pattern on a diuretic renogram was determined during the second postoperative month (if necessary), and differential renal function on renal cortical SPECT at 12 months. After the first postoperative year, all patients were followed up with yearly US.

## RESULTS

Of the 549 patients with an antenatal diagnosis of HN, 149 underwent surgery. In the remaining patients, improvement was achieved non-surgically. The follow-up and treatment algorithm are shown in Fig.1. The preoperative and postoperative findings on US, diuretic renogram, and renal cortical SPECT are shown in Table-II. The increase in renal function during the postoperative period was not significant (p>0.05). The mean preoperative follow-up time was 7 months (5–66 months). VCUG was performed in 141 (94.7%) patients. Previously, all HN patients in our nephrology department underwent VCUG, but this practice was stopped years ago. In 16 patients (11.3%), grade 1–3 vesicoureteric reflux (VUR) was identified but it spontaneously improved, without the need for treatment. One patient was operated on after 36 months of follow-up, and two patients were operated on after 5 years of follow-up. The mean postoperative follow-up time was 6 years (4–11 years). Postoperative follow-up revealed re-stenosis in five patients, pyelonephritis in five patients, lumbar hernia in three patients, and perinephritic abscess in one patient. Five patients underwent repeat pyeloplasty: three within the first postoperative year, and two during the sixth and seventh postoperative years. One of those patients underwent nephrectomy for functional loss and hypertension after five years.

**Table-II:** Pre and postoperative findings on US, diuretic renogram and renal cortical spect.

	*Preop. PD (mm)*	*Postop. PD (mm)*	*Preop. function (%)*	*Postop. function (%)*	*Preop. Wash-out pattern*	*Postop. Wash-out pattern*
Patient (n=149).	30.8 (19-64)	11.7(4-34)	38.6(15-46.)	39.2 (12-50.)	O:149	NO:111 PO:24 O:14

Preop: preoperative, Postop: postoperative, PD: pelvic diameter, O: obstructive,

PO: partially obstructive, NO: nonobstructive, MM: milimeter

## DISCUSSION

Early diagnosis, careful monitoring, and prompt surgical attention are critical to the prevention of renal damage in children with UPJ obstruction.^6^,^7^ Contrary to recommendations suggesting long-term, conservative management following an antenatal diagnosis,^8^ some authors have advocated surgical treatment of such patients at the time of diagnosis, as is the case in other age groups.^9^ In a study of patients with antenatally diagnosed UPJ obstruction who required long-term follow-up, Ulman *et al*.^4^ found that only 22% required surgery. In the present study, 27.1% of such patients required surgery.

With the widespread use of antenatal US, 54–80% of newborns with UPJ obstruction are diagnosed antenatally. Patients with an early diagnosis have better preoperative renal function than patients diagnosed at a later stage of development.^10^–^12^ The general indications for surgical treatment among antenatally diagnosed patients with UPJ obstruction are an increased degree of HN, a decrease in split renal function and an obstructive wash-out pattern. Other surgical decision-making algorithms are suggested in the literature. However, opinions vary regarding the minimum differential function indicating the need for pyeloplasty; for example, according to Josephson *et al*.^13^ and Ransley *et al*.^14^ it is 40%, while Duckett *et al*.^15^ and Koff *et al*.^16^ suggested 35% and 25% respectively. In the present study, a decrease in renal function with an increase in pelvic diameter (PD) was considered to be an indication for surgical treatment, regardless of the degree of functional reduction.

An increase in the anteroposterior diameter of the renal pelvis and a decrease in parenchymal thickness on consecutive US evaluations reflect the compliance of the renal pelvis but together are an indication for surgery when accompanied by loss of renal function. However, patients with mild HN accompanied by severe caliectasis may be candidates for surgery. In the series of Dhillon *et al.*,^17^ 6 of 585 patients (1.2%) with a PD < 20 mm and serious caliectasis (intrarenal HN) underwent surgery. Among our patients, the operative findings of three patients with PD < 20 mm, severe caliectasis, and low renal function suggested that they should be considered candidates for surgery. The washout pattern on diuretic renogram is an important component of the assessment of both obstruction and the surgical success rate, but its value in neonates and infants is controversial. Koff *et al*.^8^,^18^ reported that, among children < 2 years of age with HN, the volume of the renal pelvis is the primary determinant of the shape of the washout curve (T1/2), while expansion of the pelvic volume during diuresis changes its slope and may lead to a misdiagnosis of obstruction. All patients who underwent surgery in our series had an obstructive washout pattern preoperatively. However, Ulman *et al*.^4^ reported that a washout half time of < 20 minutes does not reduce the risk of obstruction and occurs in 10% of infants requiring pyeloplasty. We observed no such cases. Rather, an improved or recovered washout pattern was present in 90.6% of our patients in the second postoperative month. Among the 18 patients with a obstructive washout pattern in the postoperative period, US revealed an enlarged renal pelvis in five patients who underwent repeat pyeloplasty while in the other patients the pattern either remained stable or became non-obstructive or partially obstructive. According to Koff *et al.*,^8^,^18^ the surgical reduction in pelvic volume is responsible for the decrease in the T1/2 time after pyeloplasty and should not be interpreted as obstruction or its resolution following pyeloplasty. Based on our experience, we propose that an improvement in the washout pattern is an indicator of surgical success. A postoperative obstructive washout pattern may indicate stenosis when accompanied by progressive PD on US examination. Rushton *et al*.^19^ found that late stenosis does not occur in patients with a washout half time < 20 min at three months following pyeloplasty.

Although the primary goal of pyeloplasty is to preserve existing renal function, some studies have suggested that pyeloplasty actually improves renal function, as measured by renal cortical SPECT.^20^,^21^ Nonetheless, in our patients there was no statistically significant improvement in differential function during the postoperative period.

Among the patients evaluated in this study, three (2%) who had been diagnosed antenatally underwent surgery after 3–5 years due to renal functional loss and an increased degree of HN. Ulman *et al*.^4^ recommended conservative long-term follow-up in patients diagnosed antenatally with HN, emphasising the critical developmental period during the first two years of life. However, they stated that these findings should not be interpreted as implying that obstruction will never occur after two years of follow-up. In their study, all patients treated surgically were under two years of age. Our findings suggest that the follow-up period should be longer than previously proposed, which at the same time raises questions regarding patient compliance and cost effectiveness. Although previous studies have recommended performing VCUG along with Ultrasound in the postnatal period in all patients with antenatally diagnosed HN, this recommendation has not been put into widespread clinical practice.^22^ At this time, we cannot recommend the routine use of VCUG, as 4.8–11.3% of the patients reported in this series experienced mild VUR that healed spontaneously.

The need for the placement of a double-J ureteral catheter or nephrostent during surgery in patients with UPJ obstruction is controversial.^23^–^25^ In our practice, we initially preferred transpelvic transanastomotic catheters, but we recently switched to using the double-J ureteral catheter.

Following surgery, an improvement in PD may be expected over the long term. Previous sonographic evaluations of patients post-pyeloplasty noted an improvement in HN in only 38–61% of patients 6 months postoperatively, although this proportion increased to 72–91% after 12–24 months.^19^ We routinely perform pelvic reduction and in this study observed a statistically significant improvement in PD in postoperative month 6 (Table II). Some studies recommend two years of follow-up,^2^ whereas others recommend 10 or more years, because of the possible development of hypertension, giant HN, and proteinuria.^3^,^5^ Our three HN patients who were followed were operated on due to renal functional loss and an increased degree of HN after 3–5 years of follow-up. In four of the patients who underwent surgery, stricture developed during the postoperative follow-up period. Therefore these patients were operated on again, after one, three, six and seven years of follow-up. In our opinion, it is important to perform long-term follow-up of patients surgically treated for loss of renal function and an increase in the degree of HN.

## CONCLUSIONS

Pyeloplasty prevents further loss of renal function and has a low complication rate in patients with functional loss and who have shown in increase in the degree of HN. Because the condition of these surgically treated patients can deteriorate over time, follow-up should continue for 10 years after surgery. In patients who are not treated surgically, a follow-up of up to six years is recommended to monitor renal function and assess the need for surgical intervention.
